# Ten things I ‘hate’ about refinement

**DOI:** 10.1107/S2059798321011700

**Published:** 2021-11-30

**Authors:** Pietro Roversi, Dale E. Tronrud

**Affiliations:** a Institute of Agricultural Biology and Biotechnology, IBBA–CNR Unit of Milano, Via Bassini 15, I-20133 Milano, Italy; b Leicester Institute of Chemical and Structural Biology and Department of Molecular and Cell Biology, University of Leicester, Henry Wellcome Building, Lancaster Road, Leicester LE1 7HR, United Kingdom; c Department of Biochemistry and Biophysics, Oregon State University, Corvallis, OR 97331, USA

**Keywords:** refinement, restraints, correlations, bias

## Abstract

Macromolecular refinement is an optimization process that aims to produce the most likely macromolecular structural model in the light of experimental data. As such, macromolecular refinement is one of the most complex optimization problems in wide use. Macromolecular refinement programs have to deal with the complex relationship between the parameters of the atomic model and the experimental data, as well as a large number of types of prior knowledge about chemical structure. This paper draws attention to areas of unfinished business in the field of macromolecular refinement. In it, we describe ten refinement topics that we think deserve attention and discuss directions leading to macromolecular refinement software that would make the best use of modern computer resources to meet the needs of structural biologists of the twenty-first century.

## Abstract 2

Ten goals for the future development of macromolecular refinement programs are described.

## 1. Introduction

Macromolecular refinement is the crucial stage in macromolecular structure determination at which the structural model is improved and finalized. Refinement is carried out by iteratively modifying parameters in the macromolecular structural model^1^ in order to optimize a target function and arrive at the most likely structural model in view of all of the data and prior information that are available (Hughes, 1941 ▸; Diamond, 1971 ▸; Konnert, 1976 ▸; Sheldrick, 2008 ▸; Bricogne, 1997 ▸; Urzhumtsev & Lunin, 2019 ▸). As such, macromolecular refinement is part of the structural biology computational toolbox, and it is typically carried out after data processing and jointly (or rather alternating) with model building and model validation, and ends with model deposition.

The best way of dealing with incomplete information always requires the joint handling of all pieces of available prior knowledge and experimental data (Jaynes, 1988 ▸). So, ideally, every stage of the structure-determination process (and macromolecular refinement is no exception) should take place as part of the one and only Holy Grail of software development for structural biology: a monster suite of programs that is able to carry out all stages of the structure-determination process in one. The ultimate structural biology software would thus integrate a wide variety of experimental data of different types, implement error analysis and propagation within one single data structure, and bridge the gap between raw data and a validated and depositable model through one giant complex system. Within such a system, refinement of NMR ensembles fitting NOE-derived internuclear distance restraints would happen jointly with NMR data processing, crystallographic refinement would take place concurrently with X-ray diffraction data processing, and fitting of X-ray-derived models to the current version of a cryo-EM map would be carried out while optimizing particle picking, 2D and 3D class averaging and single-particle reconstruction. A blueprint for such a refinement program is described in Cherfils & Navaza (2019 ▸), where the authors propose the direct use of cryo-EM micrographs as the data for refinement and validation. In another interesting development, when refining the fit of component atomic structures into single-particle cryo-EM reconstructions, the use of a resolution-dependent atomic density function makes it possible to jointly optimize the atomic model and imaging parameters of the microscope (Chapman *et al.*, 2013 ▸). Refinement pipelines with increasing degrees of integration are also beginning to accept, in addition to diffraction images (using X-rays and electrons) and cryo-EM-derived maps, data and restraints derived using different experimental techniques [such as NMR (Schirò *et al.*, 2020 ▸) or neutron diffraction (Afonine *et al.*, 2010 ▸)].

With this all-encompassing refinement program in mind as a limiting goal, we take stock of where macromolecular refinement is at, highlight areas of (so to speak) software under-development in the field, and discuss possible directions of travel for future efforts. This article is the ideal continuation of the papers by Tronrud (2004 ▸) and Tronrud (2007 ▸) (with the former paper also accompanying a CCP4 Study Weekend introductory talk). The discussion is qualitative only and most formulae and technical implementation details are omitted. The reader who wants to learn the basics of macromolecular refinement has access to the chapter on refinement in *International Tables for Crystallography* (Ten Eyck & Watenpaugh, 2012 ▸) and a number of excellent textbooks and articles; see, for example, Kleywegt & Jones (1995 ▸, 1997 ▸), Tronrud (2004 ▸, 2007 ▸), Rupp (2010 ▸) and references cited therein.

Rather than looking at the state of the art, we try to take a step back from it all and instead focus on some of the current challenges and open questions. Few of the ideas that we discuss are truly novel; in fact, to varying degrees, many of them have been debated in the field over the years and efforts have been made to address them. What we aim at is to highlight half-forgotten and/or neglected problems, rekindling their discussion and informing current and future work devoted to bringing macromolecular refinement to convergence in the twenty-first century.

We hope that our somewhat arbitrary list of refinement-related qualms will ultimately contribute ideas towards the implementation of refinement steps/tasks in the structure-determination pipeline and maybe inspire the development of the Holy Grail of a Structural Biology Monolithic System.

## 2. The case for the continued development of software for macromolecular refinement

Historically, the development of macromolecular refinement software took place in the second half of the last century, enabling the completion of macromolecular structures initially fitted to either NMR or single-crystal X-ray diffraction data (Hughes, 1941 ▸; Diamond, 1971 ▸; Konnert, 1976 ▸; Sheldrick, 2008 ▸; Bricogne, 1997 ▸; Jaskolski *et al.*, 2014 ▸; Urzhumtsev & Lunin, 2019 ▸). We know that the field has come of age as major macromolecular refinement programs are well established and lead to the deposition of an average of 40 structures a day (https://www.wwpdb.org/; Berman *et al.*, 2000 ▸). A historical perspective on macromolecular refinement is contained in the relevant sections of the brief history of macromolecular crystallography by Jaskolski *et al.* (2014 ▸).

Scientific software development takes place within the triple selective pressure exerted by (i) the questions/challenges that are current in a field of science, (ii) the changes in the nature, quality and quantity of the data available to answer these questions/address these challenges and (iii) the opportunities and limitations of the available computer hardware. As in any field of human endeavour spanning multiple decades, progress in software development does not take place in a linear manner: rather, it is subject to fashions, suffers from inertia/legacy effects and struggles with the need of handing over both successes and open questions across generational gaps.

In the light of the above, four major aspects need to be considered when assessing the need for new refinement programs or major revisions of existing programs.

**Figure 1:**
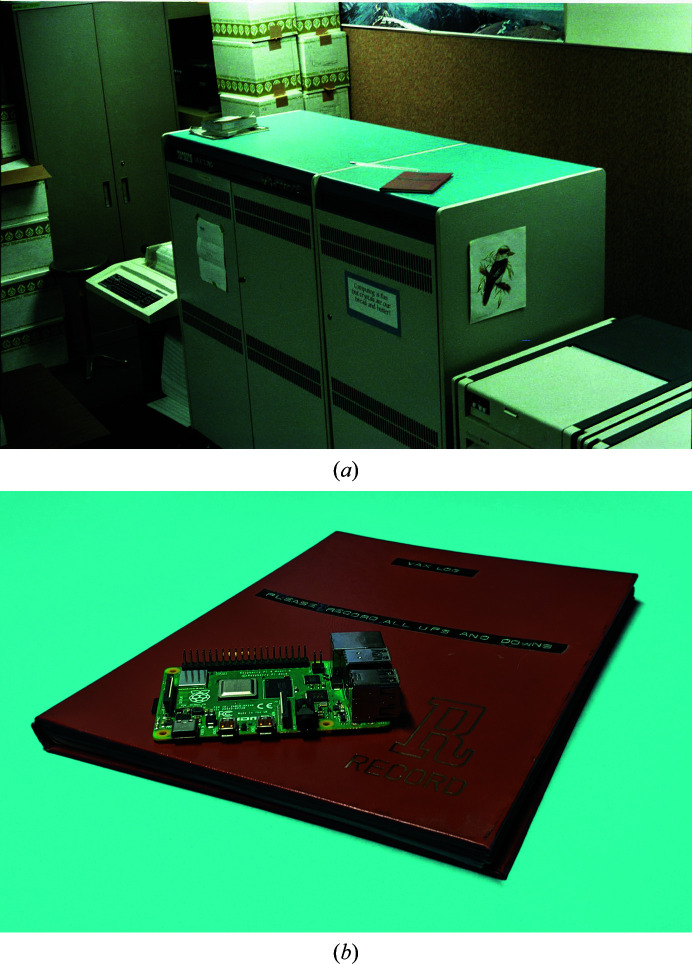
Size reduction of computers over time. In (*a*) you can see a VAX 11/780 (picture taken in the early 1980s) and in (*b*) a Raspberry Pi 4 Model B sitting on the same brown logbook as is visible in (*a*).

(i) The computing power available to the average structural biologist has increased tremendously in the last thirty years or so, and yet our refinement programs are structured in pretty much the same way. One of us (DET) bought a Raspberry Pi 4 in 2019 and ran his usual *TNT*-based benchmark on it (Tronrud *et al.*, 1987 ▸). The computer cost $50, but is 2000 times faster than the VAX 11/780 DET worked with as a graduate student and has 2000 times the amount of memory. Both machines are shown in Fig. 1 ▸. The ratio of their volumes is even larger than the ratio of their computing speeds! Despite this great increase in computing power, we are still running refinement (effectively) in batch mode, stopping for a drink and perhaps losing our train of thought in the process; it appears that on-the-fly refinement, which would update the model and maps as we build, is still some time in the future, but not for a lack of technical advances. Interesting recent developments towards real-time refinement packages are discussed in Hate #9.
(ii) Electron diffraction looks set to join NMR, cryo-EM and single-crystal X-ray diffraction as a major structure-determination technique. Yet, experimentalists in this field do not always find it easy to refine their structures using mainstream distributed software.
(iii) Historically, macromolecular refinement developed to produce models in view of two types of ‘data’: the ideal geometry on one hand and either experimental NMR data or single-crystal diffraction data on the other. Today’s refinement programs still handle only these types of data [with the notable recent exceptions of *REFMAC-NMR*, which can carry out joint refinement against NMR and X-ray diffraction data (Schirò *et al.*, 2020 ▸), and the joint refinement against X-ray and neutron diffraction data in *Phenix* (Afonine *et al.*, 2010 ▸)]. In general, though, refinement target functions that consult a variety of data are by and large still sorely missing from the majority of currently distributed refinement software. Worse, even when a multiplicity of data sets of one kind only are available, the currently available refinement programs cannot refine the macromolecular model using all of the data sets at once: for example, no existing program can utilize all of the information from a set of crystal structures for an apo enzyme and a series of inhibitor-bound complexes. This is an example of correlated observations, which are often sub-optimally handled in refinement; see Hate #5.
(iv) Improvements in macromolecular structure prediction (Senior *et al.*, 2020 ▸; Jumper *et al.*, 2021 ▸) are pushing experimental structural biology towards the determination of macromolecular complexes, so that integrative structural biology approaches are increasingly needed, consulting a variety of data types; see, for example, Russel *et al.* (2012 ▸) and Lasker *et al.* (2012 ▸). Multiple experimental techniques are necessary to generate complete structural models for such systems (Trnka *et al.*, 2019 ▸). In this respect, central to integrative biology approaches (Sali *et al.*, 2003 ▸; Russel *et al.*, 2012 ▸; Ward *et al.*, 2013 ▸; Trnka *et al.*, 2019 ▸) are the estimation of the optimal relative weight that each source of information is given in the target function, together with some means of cross-validation. Examples of software in structural biology that consult a plurality of data sets and therefore address the challenge of the relative weight that each of them contributes to the score/target function are the *PanDDA* pipeline, which actively uses many data sets of the same protein with different ligands (for example from fragment-screening campaigns) to generate high-contrast feature maps (Pearce *et al.*, 2017 ▸), and the *HODER* program in *PDB-REDO*, which also uses a complete set of homologous crystal structures to feed extra information into model refinement (van Beusekom *et al.*, 2018 ▸).

Next, we present ten of our refinement ‘Hates’. The points that we raise relate to different types of shortcomings: fundamental issues with the way that macromolecular structures are currently modelled (Hate #5), the parametrization of disordered protein regions (Hate #6) or the refinement of incomplete structures (Hate #7), which may be seen as quite distinct from implementation issues, such as the inclusion of second derivatives (Hate #2) and the handling of correlations or joint model building and refinement (Hate #3). Some of these issues are obviously intertwined, such as for example the modelling of disordered regions and the implementation of refinement of incomplete structures, or the handling of correlations, the inclusion of off-diagonal terms in the matrix of second derivatives of the target function and the choice of likelihood function. We do not have the ambition of presenting a full list, nor do we intend to rank the items in it in order of importance. Rather, we share our thoughts with the hope of encouraging those already working on some of these open challenges and perhaps inspiring others to start.

## 3. Hates

### 3.1. Hate #1. Molecular-dynamics refinement is taken too literally

In 1987, the molecular-dynamics code *CHARMM* (Brooks *et al.*, 1983 ▸) was merged with crystallographic refinement to create *X-PLOR*, and the structural biology world was introduced to simulated annealing (SA) (Kirkpatrick *et al.*, 1983 ▸; Brünger *et al.*, 1987 ▸). SA remained very popular for the next 20 years, but the only implementations generally used were along the *X-PLOR* (Brünger *et al.*, 1987 ▸) to *CNS* (Brünger *et al.*, 1998 ▸) to *Phenix* (Liebschner *et al.*, 2019 ▸) line of descent.

We believe that the SA implementations used to date in refinement have been designed with too much focus on the ‘annealing’ part of the name and not enough on the ‘simulated’ part. The main strength of SA (its large radius of convergence) has since been mostly replaced by automated model building, so it may be that worrying about the future direction of its development is a moot point. Still, SA is a powerful optimization tool and it might once again become a useful technique, especially if improved upon.

The theory of simulated annealing, in all of its fields of application, is metaphorical. Within this metaphor, parameter optimization is referred to as ‘annealing’. True annealing is a physical process in which the internal stresses of an object are reduced by heating it to a high temperature and allowing it to slowly cool. While the object is hot, its internal structure is more fluid and is able to make large changes, crossing barriers which would be insurmountable at lower temperatures. In *simulated* annealing the parameters of a model are treated as coordinates of metaphorical objects. The *objects* are allowed to possess *momentum*. The SA penalty function is considered to be a *potential energy field* which acts on the objects via *forces*.

In most uses of SA these metaphors are never viewed as actual physics. To locate a variety of examples, we performed a Google search for ‘application of simulated annealing’. We found a report in which the variables were the links of a route connecting various processing enterprises in Belarus (Grabusts *et al.*, 2019 ▸). Another example describes ‘The generation and transmission expansion planning, generator maintenance scheduling and unit commitment, reactive power planning, load forecasting and economic dispatch, and distribution systems planning and operation …’ (Huang *et al.*, 2012 ▸). The third search result was Silva *et al.* (2020 ▸), which discusses the determination of optimal parameters to model the radiation profile emitted by flames using the weighted-multi-point-source model, whatever that means. What is clear is that the parameters of the models in these three studies are not related in any way to the positions of atoms, and their use of the metaphors of temperature and potential are truly abstract.

On the other hand, the SA implementations of macromolecular refinement were designed with a very concrete interpretation of the concepts of *position*, *velocity*, *temperature* and *potential*. The identification of the metaphorical terms with actual, physical, properties makes the reuse of molecular-dynamics program code as an SA engine in refinement a tempting choice.

This is a trap! By identifying the ‘simulated’ in simulated annealing with a molecular-dynamics ‘simulation’, we lose all of the power of the metaphor at the heart of SA. Molecular dynamics can only vary the positions of atoms, and its use prevents the variation of any of the other parameters of the model. How can we vary *B* factors with molecular mechanics? Bulk-solvent parameters? Translation–libration–screw parameters? With the straightjacket of molecular mechanics imposed on our implementation of SA, we lose the ability to optimize all of these other parameter classes!

The shortcut of viewing SA as molecular mechanics risks casting the metaphor in stone. Within a molecular-mechanics framework, it becomes too easy to forget that refinement cycles are not literally steps in time, the shift in a parameter in a cycle is not literally a velocity, and the set of parameters that give the smallest value for the target function is not the lowest energy molecular conformation.

The lack of the extended metaphor in the implementation of SA in *X-PLOR* (Brünger *et al.*, 1987 ▸) restricts the scope of its optimization to varying atomic positions only. In *X-PLOR* SA runs, all other parameters are held fixed at their starting values, for no good reason.

To fully utilize the power of SA, all of the parameters of the model must be varied. The *B* factors and occupancies, as well as all of the other parameters of the model, must be given metaphorical velocities and momenta. Within this generalized framework, the metaphor of simulated annealing is a tool, not a prison.

The lack of acceptance of the metaphorical nature of SA creates other problems in the existing implementations of SA refinement. Mass is a primary concept in SA, but is inappropriately implemented in molecular mechanics-based SA. Newton’s second law (Newton, 1686 ▸) states that the acceleration of an object is calculated from the force acting upon it divided by the mass of the object. The force, in turn, is the negative of the gradient of the potential energy function, and the optimization penalty function is part of the metaphorical potential energy. In current SA refinement implementations the mass is defined as the atomic mass of the atom. What this choice ignores is that in SA we do not have a potential but a *metaphorical* potential, and therefore we have a metaphorical force, not a force. There is no reason to believe that the appropriate choice for the metaphorical mass is the actual, literal, mass of the atom!

What other choice could be made for the metaphorical mass? Mass is somewhat of a mystery in physics, but is operationally defined as the conversion factor that relates force and acceleration. It is best to think of it as the resistance of the object to changes in momentum induced by a force (a.k.a. inertial mass).

As is the case for most optimization algorithms throughout computational science, most refinement programs work by evaluating either the gradient of the target function with respect to the parameters alone, or the gradient and the matrix of the second derivatives of the same function with respect to the parameters (also called the Hessian or the normal matrix). Refinement programs using both first and second derivatives of the target function *f* calculate the shift *s* of a parameter *x* as 

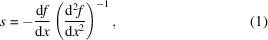

which looks very much like Newton’s second law, 

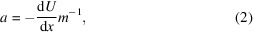

where *a* is the acceleration, *m* is the mass of the particle and *U* is the potential energy associated with the force exerted on the particle. In this one-dimensional case the metaphorical mass is equivalent to the second derivative of the target function *f* and has nothing to do with the literal mass of an atom!

When the gradient of the target function changes quickly as a parameter changes, the corresponding second derivative is large and the shift to be applied is therefore smaller than that needed for parameters whose changes have small effects on the gradient of the target function. In the metaphorical world of SA, the mass must be larger for a parameter whose changes cause large changes in the potential gradient.

This equivalence results in some unusual properties for the metaphorical mass. The first thing to notice is that the mass will almost certainly be different for different parameters, *i.e.* metaphorical directions in space. The prime example of this is when the diffraction intensities fade faster in one or two directions in reciprocal space, *i.e.* in the presence of anisotropic scattering. When this happens, the electron-density map is blurrier in the corresponding directions in real space. The changes in positions of the atoms should be more aggressive along these blurry directions, which in turn means that their masses in these directions should be smaller.

Recognizing that the metaphorical mass is equivalent to the diagonal elements of the target-function second-derivatives matrix opens many possibilities. The most obvious is the realization that simulated annealing can refine more types of parameters than just positional coordinates. As mentioned earlier, one can define the metaphorical position of the ‘atom’ to include components for the temperature factor and occupancy! The mass will then be expanded to become a tensor with different ‘mass’ components for each parameter, which will put the shifts of all the parameters on a compatible scale.

A mass tensor also would allow off-diagonal elements such as those relating an atom’s isotropic temperature factor and occupancy. There would also be significant metaphorical mass off-diagonal elements between overall crystal scale factors and individual *B* factors, as well as individual components of TLS atomic displacement factors. Off-diagonal elements of the mass tensor could be interpreted as Newtonian motion in a non-orthogonal coordinate system, but there is no need to contemplate what a mass implemented as a matrix means since all of this is just metaphor anyway. Nobody ought to be under any illusion that these calculations provide any insight into the dynamics of protein molecules: this is not the point of performing SA anyway!

### 3.2. Hate #2. Most or all of the second-derivatives matrix is ignored

By definition, the first-derivative vector of the target function points in the direction of greatest rate-of-function increase (in parameter space). The obvious way to minimize a function is to shift its parameter values in the exact opposite direction from the first-derivative vector evaluated with the current parameter values. When using this method one quickly learns that the optimum set of parameter values is not reached in one iteration. After applying the best shift along the direction of this vector, one finds that the derivative vector has changed direction and additional shifts need to be applied.

If one compares the values of the derivatives evaluated in the previous refinement cycle with the current ones, one sees that the derivatives for some parameters have changed a lot while others have stayed pretty much the same. After several refinement cycles, the parameters fall into two categories: those whose derivatives keep the same sign and those whose derivatives simply change sign each cycle. The values of the parameters in the latter class oscillate back and forth, never really settling down, while those in the first class slowly crawl along, changing a little bit more each cycle, never reaching a destination.

The second-derivatives matrix describes how the target-function first-derivative vector changes with small changes in the parameter values. With it, one can construct an optimization algorithm which can anticipate these changes and produce a parameter shift vector which, in the presence of the kind of parameter shifts discussed above, is far superior to that obtained by gradient-only methods. This is not new technology, but was invented many years before computers and is called the Newton–Raphson method or the full matrix method (Simpson, 1750 ▸).

When it comes to the choice of using first-derivatives-only optimizers, or also including the second-derivatives matrix, the refinement problem at hand ought to drive the decision, and in fact both types of optimization can be used in the same program (along with other optimizers, such as grid searches) for different refinement tasks (for a discussion of this, see Tronrud, 2004 ▸). Typically, gradient-only-based optimization has a much larger convergence radius than that based on the second-derivatives matrix, while once close to the optimal model in parameter space the second-derivatives matrix is most useful in speeding up convergence. Here, we restrict attention to refinement tasks that require the second-derivatives matrix and look at some of the issues arising from ignoring some of its off-diagonal terms (*i.e.* setting them to zero instead of computing them).

The second-derivatives matrix is a square matrix with a size of *N* × *N*, where *N* is the number of parameters in the model. For a model of a macromolecule this matrix will contain a very large number of elements, and it is no wonder that considerable effort has been invested into simplifying calculations that involve this matrix, especially considering the limited computing resources available to structural biology laboratories in the past. These ‘simplifications’ have mostly consisted of ignoring large parts of the second-derivatives matrix. The ultimate simplification is to assume that the second-derivatives matrix is an identity matrix, a matrix with ones along the diagonal and zeros for all off-diagonal elements. A discussion of these approximations and the related optimization methods can be found in Section 5 of Tronrud (2004 ▸).

As one hacks away pieces of the second-derivatives matrix, the oscillations and crawling of parameter values plagueing refinement can become worse and worse. A primary example occurs when the diagonal elements of the second-derivatives matrix corresponding to parameters that define the positions of atoms and those that define the isotropic *B* factors are assumed to be equal. Since the elements relative to atomic coordinates are actually much larger than those for *B* factors, this approximation results in the atomic positions wildly over-shifting in each cycle and oscillating, while the *B* factors hardly change at all. In refinement programs that make this assumption, these classes of parameters can never be varied in the same calculation. The refinement protocol in this case refines atomic positions while the *B* factors are held fixed. Then the *B* factors are refined while the positions are fixed. A model of a macromolecular crystal actually contains many different classes of parameters [for example anisotropic *B* factors, translation–libration–screw (TLS) parameters (Schomaker & Trueblood, 1968 ▸), bulk-solvent parameters, scale factors …] and with such an approximation/protocol each of these classes must be refined in a separate calculation. This compromise not only makes the structure of the software more complex and confuses the users, but imposes the requirement to switch back and forth between refinement of parameter classes, which in turn slows convergence, often by a great deal. To our knowledge, all programs (except for *SHELXL*, *TNT* and *BUSTER*) implement separate optimizations of positions, *B* factors, occupancies, TLS parameters and various scale factors into independent and isolated code segments. This program structure is likely to result in poor convergence both in terms of number of cycles as well as wall-clock time.

To make things worse, compartmentalization of refinement into parameter classes does not entirely solve the problems associated with the lack of off-diagonal terms in the second-derivatives matrix. The refinement of isotropic *B* factors remains difficult, because the diagonal elements for large *B* factors are smaller than those for small *B* factors. Since the majority of atoms in a molecule tend to have *B* factors on the small side they will refine stably, but values for large *B* factors will change very slowly and never reach full convergence. The slow but persistent drifting of the large *B* factors in refinement is small in each cycle and usually goes unnoticed. This amounts to the illusion of convergence rather than true convergence.

To our knowledge, no program implements refinement against X-ray diffraction images or cryo-EM micrographs directly (however, see Cherfils & Navaza, 2019 ▸): in both the fields of X-ray crystallography and cryo-EM, parameters in the data reduction and structural models have successfully been optimized in separate programs because they share very small off-diagonal terms with atomic model parameters (off-diagonal elements which already are nearly equal to zero can be ignored without a significant degradation of the speed of convergence). This is true, however, only for ‘complete’ data sets with large numbers of images. When the X-ray diffraction data set is incomplete, or the cryo-EM micrographs suffer from preferential orientation (Glaeser, 2018 ▸), data-reduction parameters (such as inter-image scale factors) will have greater ties to certain atomic model parameters. In such cases (and likely in others) it may prove useful to perform refinement of data-reduction parameters and atomic parameters simultaneously through the use of a greatly expanded second-derivatives matrix.

Ignoring differences in the diagonal elements also creates problems when refining parameters for electron-rich atoms, for example metal ions and halogens. The first derivatives of the target function with respect to the positional parameters of these atoms are also large compared with those for C, N and O atoms, which make up the majority of the unit-cell contents. Since the diagonal elements of the second-derivatives matrix corresponding to the same metal positional parameters are also relatively large, the calculated shifts are not so large. If the second-derivatives matrix is ignored, and shifts are calculated from the first derivatives alone, these atoms will always be shifted too far, resulting in an oscillation about the true position. In most programs affected by this problem, the oscillations are damped by adding stereochemical restraints between the metal and its chemical ligands. Since the ligands are composed of smaller atoms, and are usually bound by their network of geometric restraints, the motions of the metal are quite efficiently quieted. While this solution works quite well it introduces a new problem: what are the appropriate stereochemical restraints for each metal–ligand interaction? This question is difficult to answer since the geometries of metal coordination depends on the ligands.

The best way to determine the correct geometry is to determine the crystal structure, but you run into a Catch-22 situation (see Heller, 1961 ▸) if you have to restrain the metal to determine the structure in the first place!

The actual chemistry of metal sites in proteins is often unknown and therefore one cannot devise a set of restraints that are accurate. If there are experimental data of sufficient resolution to define this chemistry, they should not be interfered with by the application of inappropriate restraints. Therefore, restraints on metal-binding sites are almost invariably suboptimal and are to be avoided, unless the map is so poor that it cannot reveal the actual chemistry.

The best solution, when your data set is of sufficiently high quality to resolve the geometry around the metal atom and the density is strong enough to keep the ion in place on its own, is to leave the metal–ligand geometry unrestrained but ensure convergence by including at least the diagonal of the second-derivatives matrix.

If the data are not good enough to support the model without restraints, one is forced to make a best guess at the chemistry and impose restraints. Two important resources for the refinement of metal-containing structures are the MetalPDB database (Andreini *et al.*, 2013 ▸; Putignano *et al.*, 2018 ▸) and the implementation of algorithms that help in validating the chemical nature of a metal site (Müller *et al.*, 2003 ▸; Zheng *et al.*, 2017 ▸). Alternatively, the database of metal–ligand distances in *International Tables for Crystallography*, derived from metallorganic entries in the CSD, offers sensible values for most metal-containing protein sites refined against data whose low resolution does not allow the atoms of the metal site to be resolved fully (Orpen *et al.*, 2006 ▸).

Yet another example of a persistent problem in refinement caused by assumptions imposed on the second-derivatives matrix concerns atoms located near (or on) special positions in the unit cell. These atoms penetrate their own symmetry images. Of course, no atom can overlap the core electron density of any other atom when considered in physical space. While in actuality the symmetry of the crystal is broken at these places, the electron density being fitted is averaged by most refinement programs to enforce crystal symmetry. These atoms are ‘special’ only because their apparent overlap is with symmetry images of themselves and not with other atoms. The apparent overlap of electron density of two atoms always creates off-diagonal terms in the second-derivatives matrix, but for these atoms the off-diagonal elements relate parameters of the same atom. Atoms sitting on symmetry axes are therefore unusual in that they display large second-derivatives matrix elements between positional and atomic displacement parameters of a single atom, which otherwise are usually small. In most programs, the positional parameters of such an atom along directions perpendicular to a symmetry axis risk under-shifting, while the shifts along the axis may oscillate. The underlying cause is that an atom moving away from the axis can move further if its *B* factor can simultaneously increase. Even if both classes of parameters are co-varied, setting these off-diagonal elements to zero indicates to the optimizer that no connection should be expected. While this problem can be cured by calculating a second-derivatives matrix block for each atom, including the correction for the symmetry-related atomic overlap, most programs only provide tools equivalent to cold medicine: ignore the cause but hide the symptoms. The result is a requirement for a complex set of constraints to hold the atom on the axis and fix its occupancy. Alternatively, these atoms can be treated like metal ions and otherwise unnecessary stereochemical restraints imposed: for example by selectively switching off nonbonding restraints involving the atom on the special position (see the TNT EXCLUDE card in input in *BUSTER*); manually set distance restraints can also prevent atoms drifting from special positions or axes (also possible, for example, in *BUSTER* with NOTE BUSTER_DISTANCE SYMM). In some programs the occupancy of the atom on the special position needs setting to 1/*g*, where *g* is the site symmetry multiplicity.

To briefly conclude this section, there are quite a few other difficulties in refinement created by second-derivatives matrix approximations.

(i) Shifts in coordinate parameters of atoms connected by geometry restraints will be hampered.
(ii) It is well known that the *B* factor and occupancy of each atom are strongly correlated and the absence of off-diagonal second-derivatives matrix terms containing information on the joint changes of an occupancy parameter and a *B* factor causes slower convergence. This is exacerbated in programs that carry out alternate refinement cycles of positional, thermal motion and occupancy parameters.
(iii) The overall scale factors relating the calculated and observed diffraction intensities have off-diagonal elements with every atomic *B* factor and occupancy. Ignoring these elements results in a slow drift of all of these parameters when many cycles are performed. This drift is slowed further by separate refinement of parameter classes.
(iv) The maximum-likelihood model-error parameters are also tied to atomic parameters in the second-derivatives matrix. Unifying their refinement is more difficult since model-error parameters are best determined from the test set alone and their refinement must be isolated from the refinement of the atomic parameters (Urzhumtsev *et al.*, 1996 ▸). This requirement is thus an apparent paradox in that refinement of model-error parameters would simultaneously be driven by the free set and correlated to the refinement of the parameters driven by the working set!
(v) With *N* parameters, the first-derivative-only optimization methods used in some macromolecular refinement programs formally require *N* cycles of refinement to accomplish the work of a single full second-derivatives matrix cycle (Fletcher & Reeves, 1964 ▸). None of these programs are actually run for this number of cycles and therefore cannot possibly be considered to have converged.

The final problem that we will list is that the only proper estimation of the standard uncertainties of the parameters of the model at the end of refinement is calculated from the inverse of the second-derivatives matrix. This calculation cannot be performed without a complete calculation of the matrix (as is performed by *SHELXL*; Sheldrick & Schneider, 1997 ▸) or without performing the same refinement many times, each time using a slightly perturbed model, or even rebuilding the same model multiple times (Terwilliger *et al.*, 2007 ▸). To make matters worse, PDB records that describe uncertainties in coordinates and *B* factors (SIGATM and SIGUIJ) were actually removed from the PDB format standard at some point (moving from version 2 to version 3), even though some entries used these. Fortunately, mmCIF does allow the tracking of these values. Cowtan & Ten Eyck (2000 ▸) discussed the problems with the calculation of standard uncertainties on parameters in refined models. We are not aware that any major refinement programs (with the exception of *SHELXL*) either attempts estimation of these errors or outputs them in the PDB/mmCIF file at the end of refinement.

We do not discuss here the possibilities offered by current hardware. Suffice to argue that *SHELXL* has been calculating and storing full second-derivative matrices, at high resolution, for decades now. Clearly, this can be performed at the smaller end of the size scale of proteins, which means that larger and larger proteins will become practical as computer hardware moves on. Inverting the matrix, or worse yet eigenanalysis, is still a challenging problem, where the limitation is not storage but CPU cycles. Parallelization is a great hope and here computer clusters and GPUs would really help. Hessian-based model reduction for large-scale systems with initial-condition inputs also exist that enable efficient model reduction for large optimization problems (Bashir *et al.*, 2008 ▸).

### 3.3. Hate #3. The lack of handling of correlated observations

Refinement programs are designed with the explicit assumption that the ‘observations’ are independent of each other. Rollett (1970 ▸) discussed this assumption in small-molecule refinement programs before the first macromolecular refinement program was even a dream. ‘Independence’ cannot be assumed simply because each observation was a separate act. The relevant criterion for ‘independent observations’ is that no observation can be predicted from any or all of the others.

Do the observations in macromolecular refinement suffer from such correlations? Yes, correlated observations abound in our field! Data merging does filter out some of these correlations, but in many cases their presence has simply been ignored to the detriment of the ease of use of the software and the quality of the resulting model. It follows that good refinement programs should provide a general framework for the explicit handling of such correlations.

A number of the unresolved issues in refinement (which we combine in the one ‘Hate’ discussed in this section) all draw their ultimate cause from the lack of handling of correlations between observations. Our list includes the following.

(i) Shouldn’t we be refining against unmerged data?
(ii) Couldn’t we avoid all space-group errors by refining in *P*1?
(iii) When more than one data set is available for a given structure (including data from different polymorphs) shouldn’t all models of the structure be refined against all data sets jointly?
(iv) Couldn’t model validation statistics better than free *R* values be developed for twinned data?^2^
(v) Why don’t we routinely refine with Friedel pairs kept separate?^3^
(vi) Why is refinement in the presence of noncrystallographic symmetry (NCS) such a pain?
(vii) Why are we imposing chiral and planarity restraints when there is no mathematical justification for them?

In the following paragraphs, we briefly touch on some of the abovementioned outstanding refinement issues caused by the suboptimal handling of correlations between observations.

#### 3.3.1. An example where correlations between observations are important

The most obvious example of correlated observations in macromolecular crystallography arises when one contemplates refinement against unmerged diffraction intensities. In the case where the intensity of a particular reflection has been measured *n* times, the *n*th measurement certainly does not produce as much new information as any of the previous *n* − 1. One can indeed make a fairly reliable prediction of the *n*th measurement from the first *n* − 1 measurements. This set of *n* measurements does not contain *n* times the information of a single measurement.

A refinement program written to read unmerged data and treat them as independent observations would calculate shifts to fit each measurement in turn. A set of shifts will be calculated for the first measurement. To this will be added a set of shifts for the second measurement, then shifts for the third and fourth measurements will be calculated and summed, and so on. Since the *n* measurements are nearly the same and the calculated intensity for this reflection will be identical for all of them, the *n* sets of shifts will be nearly identical. All that happens is that the shift one would calculate for a single measurement is roughly multiplied by *n*. This would not cause a serious problem in refinement since the fraction of the shift is determined by a separate step. One would have an initial estimate for the shift that is *n* times too large, but the final shift will be cut back by a compensating factor of 1/*n*.

Problems will arise, however, because only some reflections are measured *n* times. Some are measured *m* times and their shifts will only be *m* times too large. Others will be measured *p* times and their shifts will be *p* times too large. When all of these shifts are added together and a total shift fraction is calculated, no shift will be optimal. Reflections measured many times will end up with their contribution to the shifts of the model parameters being overestimated, while those with few measurements will result in shifts that are too small.

Such refinement will not converge easily, since some subsets of data are causing oscillations while others are slowly dragging towards a solution. Current protocols avoid this problem by averaging together the many observations for each reflection. The *n* measurements become one in the data set presented to the refinement program, and the *m* measurements for another reflection also become one. In the end only one ‘measurement’ is supplied to the program for each reflection and the problem disappears, along with a lot of information encoded within the individual measurements.

To make use of unmerged data sets we need to present to the refinement program the individual measurements as well as the width of their distribution, which can be recast into a correlation coefficient. If the individual measurements (along with their relationship to each other and their correlation coefficients) are given to an optimization program, a consistent set of shifts can be calculated. How can this be done?

#### 3.3.2. Handling correlated observations

The basic least-squares equation (and the commonly used maximum-likelihood form is not much different) is 

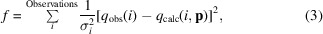

where *f* is the function to be minimized by varying the parameters of the model **p**, *q*(*i*) is some measurable quantity (either observed or calculated) and *i* is just the pointer to a particular observation. Each term is divided by the variance of the distribution of the difference between the observed quantity and the prediction of the model given the parameters **p**.

Equation (3) has no mechanism to describe a correlation between the differences for two observations. Instead, equation (3) implements an uncertainty model where there is an individual variance for each observation. The next more complex model would be expanded to include a covariance matrix, which we will call **V**. This matrix is square and its size is *N* × *N*, where *N* is the number of observations. The form of **V** is 

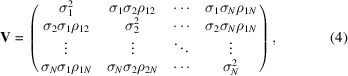

where ρ_*ij*_ is the correlation coefficient between the *i*th and *j*th residuals. In a maximum-likelihood formalism these coefficients will depend both upon the model and the observations, while in least squares they will only depend upon the nature of the observations themselves.

When the covariance matrix is substituted for the simple 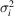 values, and the least-squares target function in equation (3) is recast into its matrix form, one obtains 

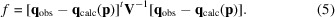

Note that the differences between the observed and calculated quantities are now represented as vectors.

Given equation (5) for *f*, how does one optimize its value? One performs the usual calculus to generate the Newton–Raphson optimization equation. We first need the gradient of the residual equation, 

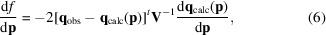

and the second derivative, 

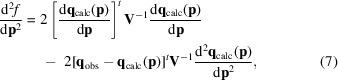

where the last term is conventionally ignored.

The shift to be applied to optimize equation (5) is the inverse of the second derivative times the first derivative, or 

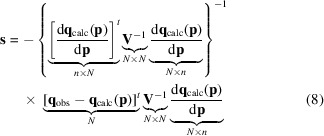

(note that the dimensions of each portion of the equation are listed below it: *N* is the number of observations and *n* is the number of parameters).

The covariance matrix **V** occurs in two places in equation (8). In the first factor it is located at the heart of the calculation of the normal matrix, which is used at the end of refinement to calculate the standard uncertainties. In the second factor it weights the importance of each discrepancy between the model and the observations in calculation of the shifts. Overall, it is obvious that proper error analysis cannot be performed without consideration of the correlations in the observations.

The enormous size of **V** makes the evaluation of equation (8) many times more difficult than the equivalent expression when **V** is assumed to be diagonal. To make the implementation of a computer program for the calculation of equation (8) feasible, some simplification of **V** is required. Such simplifications cannot be devised until the details of the correlations between the kinds of observations encountered in macromolecular refinement are made explicit.

For most types of observations **V** is very sparse. For example, in the case of unmerged data **V** becomes a block-diagonal matrix with one block for each reflection. Some of these blocks will be 4 × 4 while others are 5 × 5. These matrices are very easy to invert and matrix multiplication by the entire **V** could be performed efficiently.

#### 3.3.3. Noncrystallographic symmetry is a pain

Another excellent example of correlated observations in macromolecular refinement are those between structure factors related by noncrystallographic symmetry (NCS). Crystals with NCS were avoided whenever possible in the early days of protein refinement, until NCS was eventually recognized as tolerable and even helpful. A serious problem was first reported by Kleywegt & Jones (1995 ▸), however. They noted that at less than atomic resolution, the differences between NCS-related copies in models in the Protein Data Bank (PDB; https://www.wwpdb.org; Berman *et al.*, 2003 ▸) were larger than could reasonably be expected. The authors of refinement programs responded by including strong NCS restraints to damp out these differences. This solution, however, only treated the symptom but left the disease unchecked.

What is the disease? The primary reason that refinement against NCS-containing data means trouble is that the real-space NCS is matched by NCS in reciprocal space. Thus, the NCS relationships between the atomic positions of the several NCS copies result in relationships between the diffraction intensities of various NCS-related reflections.^4^

The simplest way to visualize the consequences of the reciprocal-space intensity relationships is to examine a special case. When twofold NCS (combined with a crystallographic twofold screw) results in pseudo-centering of the crystal diffraction pattern, alternating strong and weak intensities are observed. The strong reflections in this pattern inform on the average structure of the two NCS-related molecules, while the weak reflections provide information about their differences.

Current refinement-package implementations calculate model corrections based on the difference between the observed and calculated structure-factor amplitudes (roughly the square root of the intensity). They do not consider the individual circumstances of each reflection in this calculation. In the presence of NCS-induced pseudo-centering, the differences for the systematically strong reflections will overwhelm any influence from the differences in the weak reflections, allowing the differences between the two models to effectively be larger than the diffraction data would demand. In the absence of a correction for this effect, the distortions reported by Kleywegt & Jones (1995 ▸) are inevitable.

NCS restraints, while solving the immediate problem, are an uncomfortable solution. There have always been problems with devising a successful way to weight these restraints relative to the diffraction data. Since the NCS-related copies of the molecule in the asymmetric unit really do differ to some extent, one cannot simply tighten the weight until they are identical. The NCS weight problem is even worse, however, because the copies differ by varying amounts over the length of each NCS-related polypeptide chain, with the worst case being loops whose structures bear no similarity at all across the NCS-related copies. An additional problem of this sort arises in the case of higher order NCS, where some subsets of molecules have high structural similarity within the subset but differ much more across subsets.

A great deal of work has been performed to overcome the weighting problems created by NCS restraints (for example torsion, global and local ‘autoNCS’ strategies; see, for example, Murshudov *et al.*, 2011 ▸; Smart *et al.*, 2012 ▸; Headd *et al.*, 2014 ▸). We believe that a solution that requires 30 years of work to develop and remains problematic in practice and difficult to explain to users cannot be the correct approach. If your ‘solution’ to a problem creates yet another weight, and a weight that you cannot easily figure out how the refinement program can set, you are on the wrong track.

A new approach is needed and the direction of this approach can be found by going back to the original problem. NCS is not a problem because of a lack of information in real space, but because of the failure to build the symmetry relationships between intensities into the optimization mathematics. What is needed therefore are target functions that evaluate the likelihood of a structural model jointly against sets of NCS-related amplitudes, similar to SAD/SIRAS likelihood functions that enable heavy-atom refinement in the presence of anomalous signal and depend jointly on Friedel pair structure-factor amplitudes (de La Fortelle & Bricogne, 1997 ▸; McCoy *et al.*, 2004 ▸; Murshudov *et al.*, 2011 ▸). A first step towards the implementation of such joint likelihood functions is the treatment of refinement in the presence of tNCS described in Read *et al.* (2013 ▸). Equivalent treatments that enable refinement against sets of generic NCS-related structure-factor amplitudes (*i.e.* NCS with nonzero rotational operators) still await implementation, although an algorithm to determine the translation relating the NCS copies, any small rotational differences in their orientations and the size of random coordinate differences caused by conformational differences has been published (Read *et al.*, 2013 ▸).

While these efforts are the beginnings of understanding NCS correlations, implementing them in refinement software as special cases in no way helps the other situations where there are correlations between observations. We believe that the more general approach of implementing **V** and using the methods mentioned above to calculate the off-diagonal elements results in a much more useful tool. In the case of NCS, NCS symmetry would appear in **V** as nonzero off-diagonal elements for each set of NCS-related reflections. These elements cannot be arranged in a simple way, like the block-diagonal form for unmerged data, but certainly **V** will be very sparse.

#### 3.3.4. Polymorphs, and apo and holo structures, should be refined against multiple diffraction patterns

When solving the structures of a protein in complex with different ligands, or when in the presence of crystal polymorphism, the structure-factor amplitudes of different data sets are correlated by the underlying macromolecular structure common to all crystals. In the case of apo and holo structures, the structure-factor amplitudes of complexes in isomorphic crystals are very similar (they all belong to the same polymorph). Often the *R* value between two data sets corresponding to two different ligands is less than 15% and can be much lower. This value can be lower than the final *R* value between the model and the data set that it was refined against! Clearly these pairs of data sets are not ‘independent’ in the sense used in this section.

As in the case of NCS, when you have two, highly correlated, data sets, which you fit with two models while ignoring the correlation, the resulting models will have more differences than are justified by the data. We have become accustomed to restricting NCS differences at medium resolutions to avoid spurious differences, but we have no equivalent practice for pairs of isomorphous macromolecule–ligand complexes or for instances of the same macromolecule in a different crystal lattice environment.

Terwilliger & Berendzen (1995 ▸) was a start in that it contains a procedure for calculating maps showing only those differences demanded by the data sets.

Tronrud (1996 ▸) (an author of this paper) went further to describe test calculations of the joint refinement of up to four thermolysin–inhibitor complexes. The model in this study consisted of a single model for the N-terminal domain and another model for the C-terminal domain of thermolysin, and a unique model for the active-site region of each of the four crystals. The two domains were required to be identical in all four crystals, with the addition of a separate rigid-body transformation in each crystal. These domains were refined against all four data sets. The resulting model had slightly more than one quarter of the number of parameters of the conventional four separate models, but the *R* values were only a little larger. This joint refinement model had no spurious differences between crystals, since exactly the same model was used in all four. There were indications in the final difference maps that some additional non-isomorphism would have been justified, particularly at crystal contacts, but overall the difference maps were nearly as flat as those from refinements against individual models.

The joint refinement of isomorphous structures is very similar in nature to refinement against unmerged diffraction data. The correlations in the observations can be handled in exactly the same way, but joint refinement requires the creation of an explicit model of the non-isomorphism that relates the contents of the crystals used in the refinement. While this model may be difficult to design, it is of fundamental interest since these changes are primarily caused by inhibitor binding, and it allows one to unambiguously see only the true changes in the structure.

#### 3.3.5. Chirality restraints make no sense

In the beginning there were bond-length and bond-angle restraints. Shortly afterwards, it was observed that stereogenic atoms tended to become flatter than one observes in high-resolution crystal structures and this will sometimes progress until the atom flips and its stereochemistry is reversed. Creating a model with incorrect stereochemistry upsets some people.

This had to be stopped!

When there is a problem in refinement, the solution chosen is usually to add more restraints. Thus, the ‘chiral restraint’ was created. Of course, stereochemistry at a tetracoordinated centre is simply a two-state function. To restrain such stereochemistry you need a target function that continuously progresses from *R* to *S*. Such a function would allow the calculation of the derivatives required for inclusion in the shift calculation of the refinement program. This means that any ‘chiral restraint’ only incidentally maintains the proper stereochemistry around the central atom and is actually restraining some other property of the arrangement of atoms around it.

We can see the nature of the problem that such an additional restraint causes by examining the configuration around a C^α^ atom. The relative positions of the three non-H atoms bound to this atom require nine parameters, three of which are consumed in defining the orientation. The remaining six parameters can be defined via the three bond lengths and the three valence-bond angles. There are no additional degrees of freedom for additional restraints to limit. The ‘chiral’ restraints are imposing information which is simply a recasting of the length and angle information, and therefore is redundant.

Instead of blindly piling more restraints onto the refinement, it would be best to return to the original problem and devise a more targeted solution. The source of this problem is not a lack of information, but an incorrect description of the variability of atomic positions. In well resolved structures the positions of the atoms bonded to C^α^ do not vary isotropically. It is easier for external forces to push the atoms around the ‘equator’ of the C^α^ atom than it is to move them towards either ‘pole’. (Here, the ‘pole’ of the atom is defined as the C^α^—H bond.) This asymmetry results in greater variability around the waist of the C^α^ atom but smaller variability in what has been come to be called the chiral volume.

Analysis of this variability by looking at one angle at a time results a single standard deviation that is too restrictive in one direction and overly forgiving in the other. A convenient metaphor to illustrate this is a spider walking across a table. The relative positions of the legs are quite variable, and yet their combined motions result in the body of the spider remaining the same height above the surface. If you determine the variability of the angle between pairs of legs and generate an ensemble of spider models, ignoring the correlation in motion between the legs, some of your spider models will have their bodies below the surface of the table. You will have generated spiders with inverted chirality!

A better solution to the problem of C^α^ chiral inversion is to devise a more complete description of its bond-angle variation. The next step in elaboration from three independent angles with standard deviations is to use a 3 × 3 covariance matrix. This description introduces correlation coefficients between each pair of angles. It still includes the assumption that the distribution is normal, but has one principal difference from the conventional distribution: it allows the distribution to be wider in directions of three-dimensional space that are off-axis. For the C^α^ atom, the sum of those three angles will tend to be more restricted, while their differences will be less restricted. Chiral inversion can only occur when all three angles are larger than typical simultaneously, and this type of distribution will resist the transformation.

As an example, we performed a quick search of the PDB for well defined l-alanine residues in models from the PDB at resolutions better than 1 Å. Since alanine is the simplest proteinogenic α-amino acid with a C^β^ atom, these data should show the correlation pattern with the least complication. Analysis of the 3638 residues gave the covariance matrix, with an angle order of NC^α^C^β^, NC^α^C, C^β^C^α^C: 

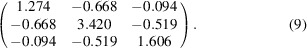

While you can see the expected result (Engh & Huber, 1991 ▸) that the NC^α^C angle has a much larger variance than the other two angles (3.420°^2^ compared with 1.606°^2^ and 1.274°^2^), there are also significant off-diagonal terms. These correlations are easier to interpret through a principal component analysis, as shown in Table 1 ▸.

The most variable of the three components says ‘when τ(NC^α^C) becomes larger than average the other two angles must become smaller’, where τ() is the IUPAC symbol for the value of a valence-bond angle (Hoffmann-Ostenhof *et al.*, 1974 ▸). The proportioning is such that the two others each pick up about half of the excess of τ(NC^α^C). This coordinated motion tends to preserve the chiral volume despite the high variability of the angles.

The least variable of the three components, at less than a third the size of the most variable, says ‘when τ(NC^α^C^β^) becomes larger the other two angles also tend to become larger at half the rate.’ In this case, when all three angles increase the C^α^ atom becomes flatter and when all three become smaller the C^α^ atom becomes ‘pointier’. The small variance of this component is what tends to preserve the chirality of the residue. The component with the intermediate amount of variability says ‘when τ(C^β^C^α^C) becomes larger τ(NC^α^C^β^) becomes smaller at half the rate.’ Since the rates are not the same, the chiral volume will change with this variation, but not as much as in the case where they act together.

When a refinement program implements valence-bond angle restraints with the proper covariance values, there will be no need for arbitrary restraints such as chiral volume or improper dihedral angles and no need for calibration of their weights. Since planarity restraints have been invented for the same reason (*i.e.* simple bond-angle restraints do not usually generate the expected planarity), the proper treatment of bond-angle covariances should eliminate planarity restraints as well.

These geometrical correlations will be largest between bond angles which share common atoms. The consequence of this is that nearly all pairs of bond angles will have no correlation with each other. The resulting **V** will be extremely sparse. Estimating the values of the correlations will be somewhat cumbersome for arbitrary molecules, but macromolecules are composed of a small number of chemical motifs that are repeated many times. One could determine the required correlation coefficients by observing the patterns in crystallographic structure databases for amino and nucleic acids. It is quite likely that they could also be deduced from long-duration molecular-mechanics simulations, but this procedure would have to be validated by comparison with large sets of crystal structures.

As we showed with the C^α^ example, modelling the correlations in valence-bond angle deviations not only allows the replacement of chiral volume and planarity restraints but also opens new opportunities for understanding, restraining and validating additional details of molecular geometry.

### 3.4. Hate #4. Ideal geometry creation is fragile

The most significant errors consistently seen in models in the Protein Data Bank (PDB) are likely to be due to poor geometry libraries. Structural biologists have come to rely on large libraries of automatically created stereochemical restraints (Engh & Huber, 1991 ▸) or are running tools that generate customized stereochemical dictionaries (Moriarty *et al.*, 2009 ▸; Schüttelkopf & van Aalten, 2004 ▸; Long *et al.*, 2017 ▸; Smart *et al.*, 2021 ▸), but do not always bother to take a critical look at either.

Stereochemical restraints are required for any refinement of a model against an experimental data set of resolution poorer than a bond length, and will also be required at atomic resolution for regions of the structure that suffer from disorder. Since these target values will not be contradicted by the experimental data (otherwise they would be unnecessary) they will appear in the final model. In this sense, stereochemical restraints are indistinguishable from actual experimental observations. Having accurate target values for these restraints is critical to producing accurate models.

Despite the importance of these target values, the process of setting their values remains difficult and error-prone, especially for users. All macromolecular refinement programs supply a large library of ideal geometries for chemical components [these days typically cast in the Crystallographic Information File (CIF) format (Brown & McMahon, 2002 ▸)]. For components that are not present in the library, there usually is a tool to automatically generate restraints.

The restraints for the 20 proteinogenic α-amino acids and the principal nucleic acid monomers have been widely used, examined by many eyes and are mostly reliable (although there are still exceptions; Moriarty *et al.*, 2020 ▸). When a user encounters a monomer or compound that is less frequently seen, they may be lucky and find it in a library supplied by the program. Failing this, they will be required to learn to use some other program which will define target values based either on small-molecule crystal structures or an empirical energy function.

We ‘hate’ the fragility of these libraries and programs. They typically have three points of failure.

(i) The chemical structure of the compound must be defined precisely for the stereochemical restraint generation to have any hope of producing correct results, but the two most used methods (either drawing the molecule in a CAD program or devising a complex and obtuse SMILES string) are cumbersome and error-prone. To make the task simpler for the user, the programs often have the option of reading a 3D atomic model of the molecule and having the program deduce the chemical structure, but this method is even more unreliable.
(ii) The second failure point is in the reliability of the empirical energy function itself. Any flaws in this function will result in inaccuracies in the restraints. Metal-containing ligands are a significant class of compounds where energy functions are often poor.
(iii) Computer programs of both varieties often contain bugs in their most complex code. Programs that utilize empirical energy functions can have errors in this part of their code, but both types of programs are susceptible to errors in the interpretation of SMILES and InChI strings.

To make matters worse, the large libraries of target values for previously encountered compounds supplied with refinement programs were probably generated via these same error-prone programs and often contain errors themselves.

As with all aspects of refinement, the final responsibility for the quality of the model lies with the user. They must inspect any set of stereochemical restraints that they apply to their model and correct any problems that they find. Unfortunately, most users do not *know* that they need to perform this check. Quite often models with gross errors in their restraints (for example a tetrahedral C atom forced to be trigonal) go unnoticed and eventually find their way into the final PDB-deposited model.

**Figure 2:**
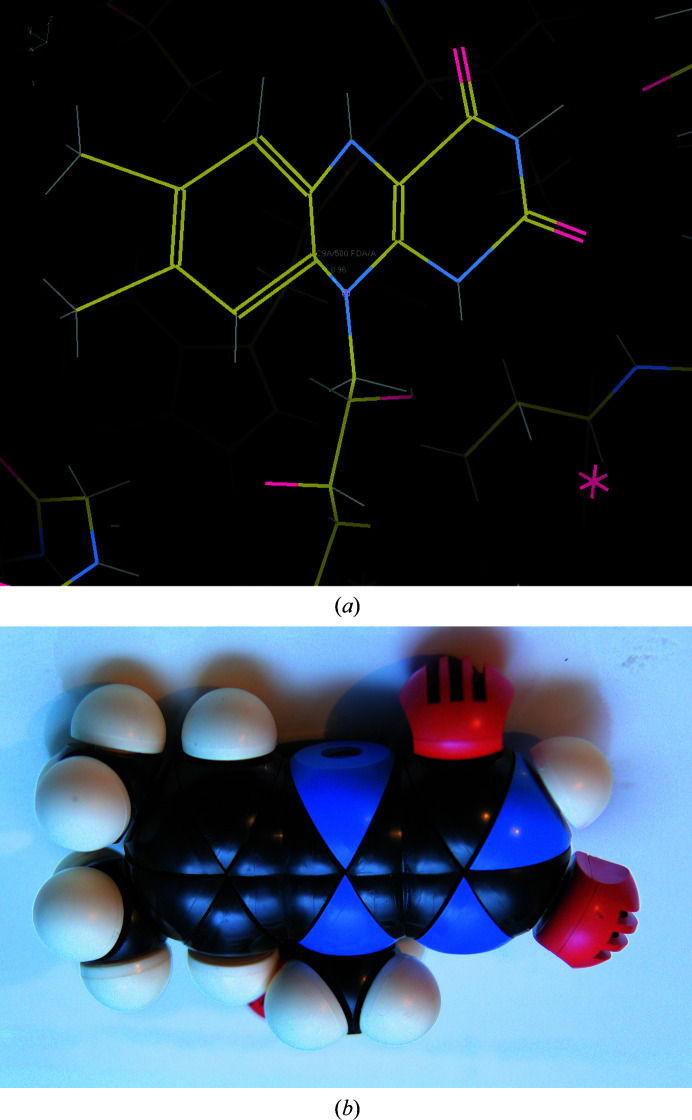
Example of the validation of automatically generated stereochemical restraints. (*a*) An automatically generated stereochemical restraint CIF was used by *Coot* to idealize the coordinates of a model of the FADH_2_ molecule. (*b*) A comparison with a CPK model of FAD easily shows the flaws in this library. It is an interesting demonstration of the limitations of ‘freshman chemistry’ that it is impossible to build a CPK model of FADH_2_ because a serious clash created by the second H atom forces a significant distortion of the ring-system plane.

Several years ago, one of the authors used the *Phenix ReadySet!* program to create restraints for FADH_2_. A model idealized with the resulting restraints is shown in Fig. 2 ▸(*a*). Clearly no refinement would have been improved by adding these restraints! While this bug was rapidly corrected, there are likely to be many more that are still around, since all complex programs contain bugs.

A final validation step needs to be inserted into the ideal value generation process in every program. As shown in Fig. 2 ▸(*b*), a comparison of the new library with some very simple and old-fashioned tools would easily reveal the error. CPK models (Koltun, 1965 ▸) contain a relatively small number of atom types and can be considered to embody ‘freshman chemistry’ level chemical knowledge. With the apparent failure of many structural biologists to use their knowledge of freshman chemistry to assess the quality of the restraints they are using, software must be written for this task.

Every ideal geometry generation program should include a final step which compares the (no doubt subtle and precise) values that it just generated with a set of very simple-minded values and possibly rejects its own faulty conclusion, before passing it on to the unsuspecting user.

### 3.5. Hate #5. The *R* values are too damn high!

In the very early days of macromolecular refinement, back in the 1970s, structural biologists were just happy to get *R* values below 30%. As computers became more powerful and the software improved, it started to become clear that creating macromolecular models that fitted the diffraction intensities within the levels of agreement routinely achieved in small-molecule crystallography was unreasonably difficult. Well, not ‘difficult’, ‘impossible’.

Grumbling began and continues to this day.

Many explanations have been proposed over the years. Many of them proceed from the assumption that there is something fundamentally different about protein crystals and the solution lies in either adding a novel kind of parameter to the atomic model or another mathematical correction to the calculation of ‘*F* ^2^’ from the diffraction image.

We believe, and this idea has certainly been floated before, that the answer is simpler. Our community has not developed a method of interpreting low-resolution images with sufficient detail.

When you compare a model in the Protein Data Bank based on low-resolution data with one based on high-resolution data, the key differences are as follows.

(i) The low-resolution model has a much lower water molecule to residue ratio.
(ii) The low-resolution model has almost no alternate conformations.

Water molecules are bound to protein atoms via hydrogen bonds, which typically have a length of 2.8 Å but can be as short as 2.3 Å. At data resolutions better than these, and in well ordered regions of the structure, water molecules appear as spherical, isolated blobs surrounded by an irregular arrangement of atoms that must be either acceptors or donors of hydrogen bonds. At worse resolutions, these features of the image will be blurred and the various players will be harder to recognize. Compounding the problem, our model-building strategies start by building and refining naked protein and *then* looking for water molecules. In this process the partners of the water molecule will be pushed by the refinement program into the unmodelled water density, obscuring its signal in the resulting difference map. Most of the current refinement programs have written into their code the goal of explaining *all* difference map features by moving the atoms in the model, despite the certainty that early models of a protein are incomplete (see Hate #9).

The lack of an explicit model of disordered fragments in low-resolution models has been so pervasive that we get into the habit of only thinking about alternative conformations at high resolution. We sometimes overlook what we all know: a data set is of lower resolution because that structure has more disorder. This disorder can occur at a number of levels (for example crystal, domain, secondary-structure element or residue), but disorder of all categories shares the same source: our sample is composed of an ensemble of molecules whose members differ from each other in conformation (Ploscariu *et al.*, 2021 ▸). While a ‘low-resolution’ model should have more variability, the models we build against low-resolution data actually have less!

To improve the fit to our data, we first need to develop robust model-building algorithms that can produce the correct hydrogen-bonding network (or ‘networks’ when alternative conformations are included) similarly to what has been performed to predict the positions of H atoms in side chains (Hooft *et al.*, 1996 ▸). These networks obviously must include buried water molecules, which are crucial for the correct placement of the surrounding protein secondary-structure elements. These internal water molecules are ‘low-hanging fruit’ because they are usually well ordered, have specific interactions with protein that can likely be categorized in some way and have an outsized influence on large parts of the protein structure. Surface water molecules, with their partial occupancies and alternative hydrogen-bonding networks, will be a much greater challenge, but are likely to have less of an effect on the backbone structure of the protein (Defelipe *et al.*, 2018 ▸).

We recognize that this strategy is not the entire solution to the problem of the high *R* values of macromolecular models. Even small proteins with little disorder and high-resolution diffraction have higher *R* values than small molecules, but even a partial solution that would bring the *R* values of most macromolecules down to this level would be welcome.

### 3.6. Hate #6. Stereochemical restraints are applied too weakly in low-occupancy regions

The weights on stereochemical restraints are generally set globally and are calibrated either by minimizing the free *R* or steering the overall r.m.s.d. from the stereochemistry library to desired values (say 0.01 Å for the r.m.s.d. on bond lengths and 1° for the r.m.s.d. on bond angles). Generally, the model ends up with tighter compliance when the diffraction data set is of lower resolution.

Some parts of any type of macromolecule, however, have more ‘disorder’ than others. This variability makes the image derived from the average of many individuals blurrier in these regions: the resolution is effectively worse in those areas. The local variation of resolution requires that the stereochemical weighting must also be local. A similar ‘local resolution’ variability is also well known in cryo-EM (Vilas *et al.*, 2018 ▸).

We typically model disorder in two ways. The most common is to assume that each atom varies in position in the individual molecules centred on a particular location with a roughly normal distribution. In this case, the *B* factor of the atom increases when there is a wider distribution. The presence of large *B* factors indicates a loss of information and the stereochemical restraints need to be relied upon more heavily.

The other type of disorder occurs when the distribution of locations for an atom has multiple centres. In this case we place an ‘atom’ at each centre, mark them with different ‘alt loc’ identifiers and set their fractional occupancies to less than one. Generally one location will be occupied much more often than the other and, in the older PDB idiom, this ‘alt loc’ is named ‘A’ while the lower occupancy site is labelled ‘B’ (this is nearly always the case, but there are exceptions in the PDB). These lower occupancy regions of the electron density or electrostatic potential will also be less informative and require higher weights on the stereochemical restraints.

Current refinement programs, unfortunately, do not vary the strength of their restraints based on these properties of the experimental data. The result is that the restraint r.m.s.d. will become unacceptable in these regions even though the global r.m.s.d. is acceptable. As an example, we looked at the model of hen egg-white lysozyme with PDB code 2vb1 (Wang *et al.*, 2007 ▸). This is an otherwise well refined *SHELXL* model based on 0.65 Å resolution X-ray diffraction data. While the overall r.m.s.d. for bond angles falls in the acceptable range, the r.m.s.d. for the 135 bond angles that include only ‘A’ atoms is 2.38°, while the same angles in the ‘B’ atoms have an r.m.s.d. of 4.63°. This r.m.s.d. is unacceptable, but is not flagged by any validation suite because the stereochemical restraints are never broken down in this way.

Refinement and validation programs should be modified to ensure that this problem is detected and prevented. Just as a 4 Å resolution model should be allowed only a very small stereochemical r.m.s.d., the high *B*-factor and low-occupancy regions of high-resolution models should be much more tightly restrained than they routinely are.

### 3.7. Hate #7. Refinement of incomplete models has been incompletely implemented

Refinement of incomplete models introduces bias, and can converge to false minima that prevent completion of the structural model (Lunin *et al.*, 2002 ▸). For one, the overall scale factors between the observed and calculated structure-factor amplitudes depend quite crucially on the model, and when large parts of the model have yet to be built the errors affecting the scale factors will deteriorate the quality of the residual maps that are crucial to build the missing structure in the first place (Afonine *et al.*, 2013 ▸).

A second related and often neglected challenge in the refinement of severely incomplete structures is that bulk-solvent models are routinely based on the complement of the macromolecular model (Murshudov *et al.*, 2011 ▸; Moews & Kretsinger, 1975 ▸; Tronrud, 1997 ▸; Blanc *et al.*, 2004 ▸; Afonine *et al.*, 2013 ▸). Thus, when large loops/domains/subunits are ordered but missing from the initial model, the corresponding calculated structure-factor amplitudes not only lack scattering contributions from these atoms, but also include a wrongly calculated contribution from the bulk-solvent model. The most commonly occurring example is indeed X-ray crystallographic refinement of initial models obtained by correct molecular replacement of a subset of the contents of the asymmetric unit: for example, incomplete models for multi-subunit complexes (Hanzal-Bayer *et al.*, 2002 ▸) or initial stages of molecular replacement in the presence of a large number of copies in the asymmetric unit (Jobichen & Swaminathan, 2014 ▸). Another often neglected occurrence of biased refinement of incomplete models is the simulated-annealing refinement of a partial structure (Hodel *et al.*, 1992 ▸).

In all of these scenarios, refinement will drive the parameters of existing atoms to fit signal coming from missing atoms, which makes the refinement of incomplete models an excellent example of a dangerous endeavour ending in disaster, with models containing as little as 40% of the asymmetric unit being deemed unrefineable in the absence of, say, experimental phase information (DePristo *et al.*, 2005 ▸). In general, the incorporation of low-resolution prior information will help in the refinement of incomplete models.

When incompleteness is due to weakly scattering ligands, solvent molecules, side chains, alternative conformations and residues both in terminal regions and in loops, *Phenix* polder maps have been implemented to help model building in maps from the refinement of an incomplete model (Liebschner *et al.*, 2017 ▸). Such maps do not strictly speaking improve the quality of phases from incomplete model refinement, but simply help the model building taking place afterwards, correcting biased phases *a posteriori*.

Model incompleteness arising from larger missing domains/subunits can be taken care of by the use of phased likelihood functions (Pannu *et al.*, 1998 ▸) or by ‘educated guesswork’: building continuous, low-resolution missing-atom distributions in regions of the asymmetric unit that are likely to contain ordered, as yet unmodelled atoms (Roversi *et al.*, 2000 ▸), a strategy perhaps reminescent of the *BYPASS* and *PLATON-SQUEEZE* estimation of contributions to the structure factor from solvent regions in small-molecule crystals (Van Der Sluis & Spek, 1990 ▸; Spek, 2015 ▸). Low-resolution distributions added to the model can contribute to calculated structure factors, mitigating some of the flaws of refinement of incomplete models and aiding model completion. Quite apart from the fact that *BUSTER* seems to be the only refinement program that offers this option, the challenges related to the overlap of such missing-atom distributions with incomplete model-based bulk-solvent masks and the difficult estimation of error models to be attached to missing-atom distributions remain to be tackled.

### 3.8. Hate #8. Could we please have a CIF definition that describes residue links?

This problem originates with the old PDB coordinate file format. In that format, a protein was split into individual monomers, but there was no means of defining the chemical links between them (other than a special case for disulfide bonds). One was left to guess where there was, or was not, a peptide bond between successive residues in a chain. Metal ligation, covalent inhibitor bonds, glycosylation, nonpeptide bond residue linkages: none of these could be defined.

The mmCIF standard (Westbrook & Bourne, 2000 ▸; Fitzgerald *et al.*, 2006 ▸) was designed to replace the PDB file format, and it was a great advance in the representation of a macromolecular structure or complex. Yet, the definition of the chemical topology of the polymers was, unfortunately, overlooked once more. The reader of a file was required to deduce, or just plain guess, which residues were connected by what kind of links.

Chemical topology formally consists of two types of data: knowledge of which residues are connected to which and the detailed chemistry of each link. Here, we are most concerned with the former. How to actually describe the chemistry and refinement restraints of a link is still an active area of research (see, for example, Nicholls, Joosten *et al.*, 2021 ▸; Nicholls, Wojdyr *et al.*, 2021 ▸) and it is too soon to formalize standardized mmCIF tag definitions.

The topology is another matter. It is rather well established that macromolecular structures are described as a collection of residues, for lack of a more general term, and these residues are bound to others via links whose types are given names. This level of information could easily be defined in mmCIF-style tags.

This linkage information is critical to the identification of the stereochemical restraints, but also for many other tasks. With a comprehensive list of links a program could easily construct a graph of the structure and deduce features such as the number of branches and closed loops in each molecule. The table would also allow software to iterate over residues of a macromolecule in a structurally relevant order, moving from one piece to another, with each step being a small movement in space. These are examples of common operations that a developer may wish to perform even though they are un­interested in the specifics of the chemistry of the links.

Yes, a program could examine the entire structure and deduce the links. The most common procedure is to find residues that are close to each other and assume that they must be chemically linked. This is, surprisingly, a very complex and error-prone procedure. It is quite common to encounter models, particularly those created by molecular replacement, where residues end up very close to each other when there was no intention to imply connectivity. With such a complex decision it is certain that different developers will write code that makes different choices for some models. It would be much more reliable for the molecular-replacement program, for example, to provide the connectivity it intends along with the rest of the information describing the model.

As long as there is no standard for encoding this information in mmCIF, every computer program has to have a way of determining, unambiguously, all of the chemical connections. The solutions that have already been created were designed without consultation or cooperation between the authors of the refinement programs, resulting in incompatibilities and frequent mistakes when transferring a model from one program to another. Anyone who participates in the CCP4 bulletin board has seen questions on this topic continuing to arise over the decades.

Currently, the only way to resolve this problem is to add data items to the mmCIF dictionary. If the connectivity problem was as simple as a list of peptide bonds, the solution would be easy. There are quite a variety of types of chemical links in macromolecular complexes and a comprehensive solution is required.

This is not the place to propose a specific set of tags for the definition of the topology of a crystal structure. We will simply list some of the requirements that these tags must meet.

The number one requirement is that every link must be explicitly and unambiguously defined. One cannot be forced to assume that successive residues are linked by peptide bonds, because sometimes they are not! Sometimes the peptide bond has been cleaved but, more rarely, the residues are linked by some other chemistry. For this reason, even something as obvious as the backbone links of protein and DNA must be explicitly listed.

Our second requirement is that both of two types of links must be definable. The first type of link connects two residues within that type of molecule, and is always present. This type of link would be defined in the ‘entity’ level. Placing the link definition there reduces the number of times that it needs to be mentioned and reduces the possibility of omitting it in some chains. This type of link includes the polymerization links, as well as internal disulfide bonds, metal-ion ligation, glycosyl­ation and covalently bound inhibitors.

The other kind of link connects between two STRUCT_ASYM, *i.e.* actual chains. These may connect two chains of the same ‘entity’ type, but a key difference is that a symmetry operator and potentially unit-cell translations must also be specified. While the chemistry of these links are of identical chemistry to the other type, these include intermolecular disulfide bonds and other forms of cross-linking.

Link definitions that only specify two residues should be avoided. Links that involve atoms in three or more residues are not uncommon, particularly those involving bound ions.

The lack of the ability to define these links in mmCIF prevents the convenient interchange of this information between computer programs from different macromolecular refinement program developers. Last but not least, this shortcoming of mmCIF also limits the possibility of merging model-building and refinement code into a single application, since communication between two programs using incom­patible descriptions of macromolecular topology will require a complicated software ‘shim’ at their interface.

### 3.9. Hate #9. Refinement has not disappeared

In the early days of macromolecular refinement, one could just barely run refinement on a $200 000 computer and just barely perform model building on a specialized 3D graphics computer. The work was divided up between these two computer types and the paradigm of isolated ‘refinement’ and ‘modelling’ software was established. People with very different programming skills gravitated to one type of software or the other, and each evolved over the decades with, at most, loose integration.

Already at this time, however, it was clear that hopping between computers and software was annoying, and this was believed to be a temporary situation. In a project that one of the authors (DET) worked on, he had to wait nine hours for the Digital Equipment Corporation VAX 11/780 computer (see Fig. 1 ▸) to complete the refinement before being able to move on to the next round of model building; that is, unless the computer was also working on a colleague’s refinement job. He calculated that if a computer were available that ran 500 times faster, this interruption would be reduced to a few minutes. At that point it would make sense to merge the refinement function into the model-building program and greatly simplify the entire process.

In 2019 a Raspberry Pi 4B was tested using a *TNT*-based (Tronrud *et al.*, 1987 ▸) benchmark and was shown to run about 2000 times faster than that 1980 vintage VAX 11/780. It also contained 2000 times the RAM. The Raspberry Pi cost $50, which is 1/3500 of the price of the VAX (not considering inflation). Macromolecular crystallographers do not usually perform their computations on Raspberry Pi computers, but certainly have much more powerful computers at their disposal.

Despite this enormous increase in computer power, our field still has programs that specialize in model building and quite separate programs that specialize in model refinement. The refinement/model-building integration achieved so far is very sketchy. The internal data structures of the two appli­cations are almost always of incompatible design, resulting in two descriptions of the model in RAM, with the requirement for constant translation and synchronization. The arrangement of atoms in the model is fairly easy to handle, but there seems to be ongoing difficulties with the implementation of stereochemical restraints and even the means of defining the connectivity of residues within the molecule (see Hate #8). When the user shifts their attention from model building to refinement the transition is not only jarring, but often requires significant manual intervention just to make it work.

Computers now have sufficient power that, with proper software design, refinement should simply disappear from the user’s consciousness. While the user is looking at a residue thinking of what to do next, the computer should be working in the background to optimize the previous changes and produce a new map on the fly. Since each update to the model only affects a small fraction of the total model, it seems likely that algorithms could be developed to further speed up this calculation. The calculation of first and second derivatives of the crystallographic likelihood function might be quicker given that the vast majority of atoms are unchanged from when the derivatives were last calculated. It might also be quicker to solve for the parameter shifts if one started with the previous solution from a nearly identical model.

These algorithmic changes are mere speculation, but the computing power certainly exists to make a computer program with tightly coupled model-building and refinement tools. In recent years the first steps have been taken. In *ISOLDE* (Croll, 2018 ▸) the model is continually improved via empirical energy optimization along with real-space refinement, with the image and difference map being updated as the atoms are moved. The most recent version of *Coot* (Casañal *et al.*, 2020 ▸) includes an option for updating the *B* factors using shift refinement (Cowtan *et al.*, 2020 ▸) and the electron-density maps are updated afterwards.

These first steps towards the assimilation of refinement into model-building software demonstrates that the total elimination of refinement as a standalone process is finally on the horizon.

### 3.10. Hate #10. Refinement never ends

Last year, one of us (DET) was looking at a model of human angiotensin-converting enzyme 2 (ACE2, the main SARS-CoV-2 receptor; Hofmann *et al.*, 2005 ▸; Zou *et al.*, 2020 ▸) due to its sudden importance. This enzyme is a zinc-containing endopeptidase and it brought to DET’s mind his work long ago on thermolysin. He pulled up from the PDB the reference crystal structure used in Brian Matthews’ laboratory (PDB entry 8tln). It was immediately apparent from the electron density that the active-site residue Tyr157 should have been built with a second conformation, the peptide bound in the active site is not simply Val-Lys but is sometimes Gly-Val-Lys, the zinc ligand Glu166 should have a second conformation and there even is a low-occupancy second Zn atom! This is the same crystal form solved in the Matthews laboratory back in 1972 and models of its structure have been evolving ever since. A high-resolution procession photography data set was collected around 1980 and Meg Holmes created the first refined model (Holmes *et al.*, 1983 ▸). About ten years later, it was realized that this was not an apo crystal but contained the above-mentioned Val-Lys dipeptide. This discovery triggered the collection of a new data set on oscillation films and, through extensive model rebuilding and refinement by DET, the creation of the present model with PDB code 8tln (Holland *et al.*, 1992 ▸). Now, nearly 50 years after the initial crystal structure was determined, it is clear that the refinement is still far from complete.

You’ve got to ‘hate’ that!

## 4. Dulcis in fundo

Both of us, having been involved in the development of refinement programs, fully appreciate the tremendous achievements which are embodied in the current packages. The improvements in user interfaces, addition of automation and enormous expansion of knowledge about the chemical structure restraints of literally thousands of compounds have been far beyond what anyone dreamed of 30 years ago. We hope that you have found our little list of what yet might come into being useful. Software developers enjoy battling difficult challenges, and in tackling problems such as those in our list macromolecular refinement developers will clearly continue to meet the task. 

## Figures and Tables

**Table 1:** Principal component analysis of the distribution of bond angles about the C^α^ atom of alanine residues

Variance (°^2^)	Components
3.72	−0.25τ(NC^α^C^β^) + 0.94τ(NC^α^C) − 0.22τ(C^β^C^α^C)
1.60	−0.44τ(NC^α^C^β^) + 0.09τ(NC^α^C) + 0.89τ(C^β^C^α^C)
0.98	0.86τ(NC^α^C^β^) + 0.32τ(NC^α^C) + 0.40τ(C^β^C^α^C)

## Footnotes

1 As well as atomic parameters, this structural model of course includes various overall scaling factors, bulk-solvent parameters, twin fractions *etc.*

2 The free *R* is lower with twinned refinement even if the crystal was not twinned.

3 We note here that SAD and SIRAS likelihood functions are implemented in *REFMAC*5 and *Phenix* (Murshudov *et al.*, 2011 ▸; McCoy *et al.*, 2004 ▸) and that the latter refines, by default, against both *F* ^+^ and *F* ^−^ if they are present in the reflections input file.

4 These relationships are, incidentally, those visualized in self-rotation function plots (Rossmann, 1972 ▸) (in the case of the rotational part of NCS) and give rise to strong non-origin peaks in the native Patterson (Patterson, 1935 ▸) in the case of translational NCS (tNCS).
